# Menstrual management among adolescent girls in Uttar Pradesh, India: An examination of interpersonal and mediated communication as delivery mechanisms for practical guidance

**DOI:** 10.3389/frph.2022.1025376

**Published:** 2023-01-09

**Authors:** Suzanne J. Block, Michael K. Hauer, Alex Ezeh, Suruchi Sood

**Affiliations:** Dornsife School of Public Health, Drexel University, Philadelphia, PA, United States

**Keywords:** menstrual health and hygiene management, practical guidance, social and behavior change communication, sexual and reproductive health, adolescent health, gender equity, human rights

## Abstract

**Background:**

Adolescent girls' right to achieve menstrual health and hygiene management (MHHM) healthfully is yet to be realized. One reason is the lack of practical guidance on the procurement, use, and disposal of menstrual products. This study defined interpersonal practical guidance (IPG) as face-to-face communication and mediated practical guidance (MPG) as social and behavior change communication (SBCC) intervention-driven communications through print and media materials. We examine the impact of these two delivery mechanisms of practical guidance on adolescent MHHM knowledge, attitudes, and practices (KAP) following an SBCC intervention in India.

**Objectives:**

To understand how IPG and SBCC-driven MPG independently influence adolescent girls’ KAP on the procurement, use, and disposal of menstrual products and whether those who receive both IPG and MPG have better KAP related to the procurement, use, and disposal of menstrual cloth or sanitary pads than those who receive only one form of practical guidance or none at all.

**Methods:**

Adolescent girls' questionnaire responses from GARIMA's case-comparison evaluation were analyzed using Stata/SE 17 (*n* = 2,384). Girls were matched on sociodemographic and socioeconomic variables. *χ*^2^ analysis examined relationships between sociodemographic, practical guidance, and KAP variables. Multivariate logistic regression assessed associations between practical guidance and KAP variables.

**Results:**

There are significant associations between adolescent girls' KAP depending on whether they received IPG, SBCC-driven MPG, or both. IPG and MPG delivered together has greater odds of predicting correct menstrual management KAP than when delivered separately. These effects were most notable for adolescent girls' knowledge and practices related to using and disposing of menstrual hygiene products.

**Conclusion:**

There is urgent need to create positive, sustainable changes to address menstrual health and hygiene management. This study introduces an innovative approach that utilizes interpersonal and mediated communication as mechanisms to deliver practical guidance on menstrual management. Future interventions should implement and evaluate to better understand the role of practical guidance in ensuring all women and girls are prepared to confidently manage their menstrual health.

## 1. Introduction

Menstrual health and hygiene management (MHHM) is a sexual and reproductive health and human rights issue that has received increased recognition in recent years (1, 2). Despite this recognition from the global community, menstruation remains stigmatized and neglected in many parts of the world (2). In turn, adolescent girls are left unprepared with inadequate knowledge, attitudes, and practices (KAP) to manage their menstruation (3).

MHHM encompasses the needs of menstruating women and girls, ranging from the safe and hygienic procurement, use, and disposal of products to systematic factors that influence one's right to complete social, mental, and physical well-being (2). Menstrual hygiene management includes the “use of clean menstrual management material to absorb or collect blood that can be changed in privacy as often as necessary for the duration of the menstruation period, using soap and water for washing the body as required and having access to facilities to dispose of used menstrual management materials” (2). Systematic factors that may serve as barriers or facilitators to one's menstrual health and hygiene include access to timely and accurate information, services and materials, and supportive environments (1, 2, 4).

One barrier that persists for adolescents is the lack of practical guidance on menstrual management (1, 5, 6). Practical guidance helps to ensure adolescents know how to safely and hygienically procure, use, and dispose of materials; understand how to manage pain and other menstrual symptoms; alleviate discomfort; and know when to seek care (1, 6, 7). It is crucial to deliver this practical guidance as a foundational step to improve their KAP and increase their confidence and abilities to make informed decisions (1). Otherwise, adolescent girls will remain unaware and ill-equipped to address other barriers to effective menstrual management. Accordingly, interventions should start with understanding how practical guidance can be delivered and its influence on KAP related to the procurement, use, and disposal of menstrual products to ultimately achieve better menstrual management.

This study examines the impact of providing practical guidance through different forms of communication exposure among adolescent girls in the GARIMA, a social and behavior change communication (SBCC) intervention in Uttar Pradesh, India. GARIMA aimed to “break the culture of silence around menstruation by initiating dialogue, sustaining conversations with peers, family, and community and thereby influencing change to normalize menstruation” (8). Communication exposure occurred through face-to-face interpersonal communication (IPC) or mediated communication from SBCC print and media materials. This study defines interpersonal communication as interpersonal practical guidance (IPG), and the SBCC intervention's print and media materials as mediated practical guidance (MPG). We aim to understand how IPG and SBCC-driven MPG independently influence adolescent girls' KAP on the procurement, use, and disposal of menstrual products and whether those who receive IPG and MPG together have better menstrual management KAP. To our knowledge, no studies have explicitly examined interpersonal or mediated communication individually or together as mechanisms for practical guidance in India. To address this gap in the literature, this study sought to answer two research questions:

1. How do IPG and SBCC-driven MPG independently influence adolescent girls' KAP?
2. Do those who receive both IPG and SBCC-driven MPG have better KAP related to the procurement, use, and disposal of menstrual cloth or sanitary pads than those who receive only one form of practical guidance or none at all?

## 2. Background

### 2.1. The current state of menstrual health management in India

Menstruating women and girls make up a quarter of India's population, yet there is a culture of silence surrounding MHHM (9). The culture of silence negatively impacts adolescent girls' menstrual management KAP surrounding the procurement, use, and disposal of absorbents. Menstrual health disparities vary across Indian states and regions and are likely worse in northern Indian states, such as Uttar Pradesh, than in southern Indian states (9). Depending on where they live, adolescent girls lack access to information and cannot develop the skills needed for this natural occurrence (9, 10). Overall, a reported 71% of girls did not know about menstruation before menarche (11) and one study in the northern city of Jaipur, Rajasthan, found adolescent schoolgirls reported experiencing feelings of shock, fear, anxiety, guilt, and frustration (9). Additionally, in the northern state of Uttar Pradesh, which accounts for 19.3% of all adolescent girls living in India, there is also high levels of gender inequity that exacerbates negative menstrual health experiences (12). These negative experiences reflect the stigmatization and view of menstruation as a taboo women's issue, with most mothers and grandmothers perceiving menstruation as dirty (9). Moreover, mothers, grandmothers, other female family members, and peers that serve as key information sources share information that is often inadequate or too late (6, 9).

There are also other barriers women and girls can face that compromise their ability to procure, use, and dispose of their menstrual products safely and healthfully. For instance, many cannot afford to procure products, with 70% saying that their families cannot afford to purchase or consistently access sanitary products, and 88% use household items such as cloth or hay to serve as sanitary products (9). However, these household materials are often not cleaned properly because of poor access to soap and clean water, low levels of knowledge and awareness, or stigma and restrictions (10). Women and girls also lack access to adequate toilets and private bathing facilities at home, school, and in the community to manage their menstruation (9). The lack of access to facilities impacts their ability to sanitary use and disposal of products (13).

These systematic barriers lead to poor MHHM outcomes and impact their human rights and achievement of several Sustainable Development Goals (SDGs: 3, 5, 8, 12) (6, 10). Steps must be taken to achieve gender equity to ensure all women and girls have the right to manage their menstruation with dignity and respect.

### 2.2. Conceptual model

**Figure 1:**
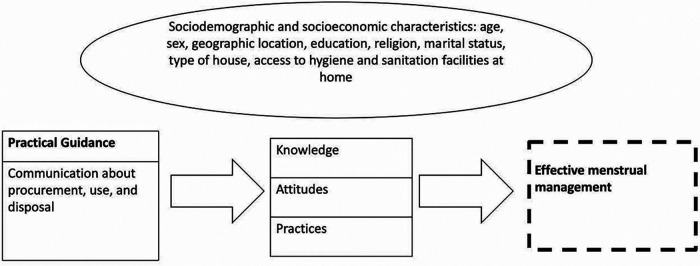
Menstrual management practical guidance conceptual model.

The conceptual model displayed in Figure 1 was developed for this study to depict the relationship between practical guidance on the procurement, use, and disposal of menstrual products, KAP, and effective menstrual health management and to highlight key sociodemographic and socioeconomic characteristics. It reflects relationships between critical menstrual management variables, which must be addressed to achieve the long-term goal of effective menstrual health management among adolescent girls. This study focuses on the association between practical guidance and KAP. The long-term goal of effective menstrual health management is featured in the conceptual model but was outside the scope of this study. This is depicted with the dashed arrow leading to the dashed box.

Communications strategies are a promising approach to promoting better MHHM (14, 15). They have been shown to increase knowledge, shift attitudes, and change behaviors (14, 16, 17). Knowledge is the concrete and interpretative information that facilitates an informed understanding of the benefits of taking action (18). Attitudes can be defined as the behavior's affective evaluation performed by the individual (19). This ability to understand attitudes toward a behavior can help predict whether that behavior takes place. Moreover, how information is communicated influences knowledge and attitudes (20). Finally, practices comprise the desired procurement, use, and disposal behaviors for effective menstrual management. Behaviors are inseparable from health (21). Overall, those with better KAP can combat negative health outcomes and support effective menstrual management (3, 22, 23).

Sociodemographic and socioeconomic factors are also essential to consider. These sociodemographic and socioeconomic factors can influence the reception of practical guidance and subsequent knowledge, attitudes, and practices. In this study, the examination of factors such as age, sex, geographic location, education, religion, marital status, and access to hygiene and sanitation facilities are included.

### 2.3. Practical guidance for menstrual management

The majority of MHHM interventions in India entail increasing awareness and promoting access to sanitary products (9). However, it is crucial to understand how practical guidance impacts KAP related to the procurement, use, and disposal of menstrual products to promote effective menstrual management. Practical guidance is meant to ensure menstruating individuals understand how to manage pain and other menstrual symptoms, alleviate discomfort, know when to seek care, and safely and hygienically procure, use, and dispose of materials (1, 6, 7). As a result, menstruating individuals have increased confidence and can make informed decisions (1). Alternatively, without this practical guidance, adolescent girls remain ill-equipped to address other determinants of MHHM. This communication should also start early and occur often to improve the timely delivery of accurate information passed from key sources of information to adolescent girls.

### 2.4. Social and behavior change communication

Social and behavior change communication (SBCC) is a holistic framework that systematically applies theory-based, research-driven communication strategies to stimulate change related to social norms and behaviors, knowledge, attitudes, and practices (24). Additionally, SBCC interventions recognize the necessity of effective communication at each level of the social-ecological model (SEM) (25). Strategies include interpersonal communication (IPC), mediated communication through print and media materials, and more.

IPC is “the tailored exchange or sharing of information, thoughts, ideas, and feelings between two or more people to address behavioral determinants of health. It is influenced by attitudes, values, social norms, and the individuals’ immediate environment” (26). IPC is an underused strategy for MHHM interventions in India despite the need for improved dialogue and support to address psychosocial and physical health concerns (15, 27). Alternatively, mediated communication is non-face-to-face communication that uses platforms such as television, film, radio, print, telephone to disseminate health messages (28). Leveraging mediated communication allows active and passive exposure to an intervention's messages and stimulates positive behavior changes. Both can also be incorporated into SBCC interventions that promote systemic changes to improve community health and well-being.

A study in two Assam districts that assessed SBCC on community members’ KAP regarding adolescent sexual and reproductive health, including menstrual health, was found to be effective (29). Another study examined an evolving SBCC framework applied in few districts in Maharashtra intended to empower, advocate, and improve menstrual health KAP and menstrual product availability and accessibility (15). They found the silence surrounding menstruation was broken. Pre- and post-menarche girls were finally engaging in dialogue, there was increased awareness, and positive changes in hygiene practices were anticipated. The authors also concluded that IPC incorporation could create sustainable changes that improve MHHM. India's Ministry of Health and Family Welfare also identifies communication efforts as fundamental because they have already played a crucial role in advancing other health and development goals in India (24). As such, SBCC interventions are positioned to address cross-cutting issues such as menstrual health in India. Specifically, IPC can serve as a mechanism to deliver practical guidance (30) alongside SBCC-driven mediated communication. IPC and mediated communication can therefore be redefined respectively as interpersonal practical guidance (IPG) and mediated practical guidance (MPG).

#### 2.4.1. GARIMA: An SBCC intervention

This study focuses on GARIMA, an SBCC intervention intended to break the silence surrounding menstruation (8). It was implemented over three years (2013–2016) in three districts of rural Uttar Pradesh, India: Mirzapur, Jaunpur, and Sonbhadra. Baseline data from these districts found that 90% of post-menarche girls were unaware of the necessity of washing menstrual cloths, and 86% felt unprepared, and 70% had low self-confidence (31). Other studies have also found low levels of MHHM KAP in Uttar Pradesh (31). With this, the 2015–2016 National Family Healthy Survey (NFHS) in India reported that 57.7% of women aged 15–19 years in Uttar Pradesh used a hygienic method like cloth or sanitary napkins for managing their menstruation, and 48.2% of women aged 15–24 living in rural areas of Uttar Pradesh used a hygienic method (32). The 2019–2021 NFHS found 70.5% of women aged 15–19 years and 68.2% of women aged 15–24 years living in rural areas used hygienic methods (33).

GARIMA implemented a communication package that reached approximately 64,000 adolescent girls (8). The package included interpersonal and mediated communication messages and materials (34). IPC components included peer educator-facilitated girl group meetings, peer educator- and health worker-facilitated one-on-one discussions, mothers' group meetings, and small fathers' groups. Mediated communication included girl group film and video watching, storybooks, and posters.

## 3. Materials and methods

### 3.1. Study design and sampling frame

GARIMA underwent a case-comparison evaluation in three districts in Uttar Pradesh, India, between December 2017 and January 2018 (8). The intervention villages were purposively selected, but adolescent participants were chosen randomly within these villages. The villages were matched on sociodemographic and socioeconomic characteristics. There were 240 villages in total (120 comparison and 120 intervention). The evaluation's pretested questionnaire assessed IPC and KAP. GARIMA measured IPC exposure in intervention and comparison groups because IPC could occur naturally through family members, peers, teachers, and other professional and non-professional acquaintances. Accordingly, IPC could be informal, non-evidence based, and can provide inconsistent information. However, only girls in the intervention villages had received mediated communication from GARIMA that was consistent and accurate information on the subject matter. As such, girls in the intervention villages received mediated communication through GARIMA and, when indicated by their questionnaire response, may also have engaged in IPC. Girls in the comparison village did not receive mediated communication but could have engaged in IPC if they stated this on their questionnaire. More detailed information on the program evaluation can be found in previous publications (8, 34).

### 3.2. Data collection

The present study utilized data collected from the pretested questionnaire distributed during the evaluation of GARIMA (8). This study focused on adolescent girls' questionnaire responses on interpersonal practical guidance and KAP related to the procurement, use, and disposal of menstrual products. Responses from 2,384 adolescent girls (*n* = 1,162 comparison and *n* = 1,222 intervention adolescent respondents) are included. The selected questions were based on their relevance to the research question and fit within the conceptual model. The questions are displayed in Table 1.

**Table 1:** Selected questions from the original SBCC intervention questionnaire (*n* = 13).

Category	Question	Type of question
Interpersonal Practical Guidance (IPG) (*n* = 3)	Where to get menstrual cloth or sanitary pad? – Have you had any discussion with anyone, or has anyone ever had a discussion with you?	Procurement
	How to use a menstrual cloth or sanitary pad? – Have you had any discussion with anyone, or has anyone ever had a discussion with you?	Use
	How to dispose of menstrual cloth or sanitary pad? – Have you had any discussion with anyone, or has anyone ever had a discussion with you?	Disposal
Knowledge (*n* = 2)	How many times should a menstrual cloth or sanitary pad be changed in a day?	Use
	How should a used menstrual cloth or sanitary pad be ultimately disposed of?	Disposal
Attitudes (*n* = 4)	It is very embarrassing to arrange for sanitary pads every month.	Procurement
	It is very inconvenient to arrange sanitary pads every month.	Procurement
	It is necessary to expose the washed menstrual cloth to direct sunlight.	Use
	It is necessary to burn or bury the used menstrual cloth or sanitary pad.	Disposal
Practices (*n* = 4)	How do you store menstrual cloth or sanitary pads?	Use
	How do you wash your menstrual cloth?	Use
	Where do you dry your menstrual cloth?	Use
	How do you ultimately dispose of used menstrual cloth or sanitary pads?	Disposal

### 3.3. Variable operationalization

#### 3.3.1. Practical guidance

We define interpersonal practical guidance (IPG) as face-to-face IPC and SBCC-driven mediated communication as mediated practical guidance (MPG). IPG among comparison and intervention groups was examined with three dichotomous questions (Yes/No) on whether adolescent girls had discussions with others on how to procure, use, and dispose of menstrual products (Table 1). If adolescent girls in the comparison group answered “yes” to these questions, they were deemed to have received IPG through non-intervention-related face-to-face interpersonal communication. Those in the SBCC intervention group who answered “yes” to these questions received IPG alongside MPG from their additional exposure to SBCC-driven mediated communication. Interpersonal and mediated practical guidance on procurement, use, and disposal were explored in relation to KAP.

#### 3.3.2. Knowledge, attitudes, and practices (KAP)

Knowledge, attitudes, and practices were the dependent variables. The original Likert-scale or multiple-choice KAP questions were dichotomized into binary variables to account for skewed distributions of responses.

Knowledge questions on the use and disposal of products had initially been multiple choice. Responses were recoded as correct or incorrect. For knowledge on how many times menstrual cloth or sanitary pads should be changed, answers were deemed correct if they selected “three or more times a day” (34) in 24 h. For knowledge on the disposal of products, answers were deemed correct if they chose to burn the absorbent, bury it in a pit (30), or store it and then take it to school (34). The original SBCC intervention focused on education on the ultimate disposal of products rather than temporarily correct solutions prior to their ultimate disposal, such as storing them in a dustbin (35). Additionally, menstrual products included sanitary pads or menstrual cloths. Menstrual cups were not identified as a menstrual product used during the original SBCC intervention's formative research and were therefore not discussed during the intervention or included in the questionnaire.

Attitude questions on product procurement, use, and disposal were originally on a 5-point Likert scale and recoded as agree or disagree. The respondents who selected “neither agree nor disagree” to the attitude questions were dropped because they represented less than 10% of the total responses.

The practice questions on using and disposing of products were recoded as correct or incorrect. Correct practices for use included always washing it with soap and water or another disinfectant, drying it outside in direct sunlight (30), storing cloth or pads with other washed clothes of daily wear, using a new cloth or pad every time (34), or storing it in a clean place (30, 35) or cupboard (34). Correct practices for disposal were burning the absorbent, burying it in a pit (30), or storing it and then taking it to school (34).

#### 3.3.3. Control variables

Additional variables were included in determining if relationships remained between IPG, MPG, and KAP after controlling for various sociodemographic and socioeconomic characteristics. These variables were age, district, type of house, education, whether there was a toilet at home, and whether there was a private bathing and changing place at home. Age was collapsed from eight levels into three, education was collapsed from four levels into three, and religion and marital status were collapsed into two levels from three. All other control variables were left unchanged.

### 3.4. Data analysis

All analyses were conducted using Stata/SE 17.0. The sample consisted of 2,384 participants, some of which received the intervention (*n* = 1,222) and some were assigned for comparison (*n* = 1,162). Proportions were calculated for sociodemographic and socioeconomic variables and the practical guidance and KAP responses (Table 2). These results include overall responses and separate responses within each sample group to allow for comparison. For the bivariate and multivariate analyses, the responses of comparison and intervention group girls are classified into four categories for appropriate comparisons: (1) None- comparison group girls who did not receive any IPG or MPG, (2) comparison group girls who received non-intervention-related IPG, (3) intervention group girls who only received MPG, and (4) intervention group girls who received IPG and MPG.

**Table 2:** Proportion of sociodemographic and socioeconomic characteristics among adolescent girls and their MHHM responses on procurement, use, and disposal variables.

Characteristics	Overall *n* = 2,384 (%)	Comparison *n* = 1,162 (%)	Intervention *n* = 1,222 (%)
Age (years)			
12–14	24.3	24.2	24.4
15–17	47.1	46.8	47.3
18–19	28.7	29.0	28.3
District			
Jaunpur	36.5	35.5	37.4
Mirzapur	33.3	32.7	33.9
Sonbhadra	30.2	31.8	28.7
Type of house			
Kucha	47.4	43.0	51.5
Semi pucca	24.2	25.2	23.2
Pucca	28.5	31.8	25.4
Religion			
Hindu	97.2	97.5	96.8
Other	2.9	2.5	3.2
Education			
Primary school/No formal education**^a^**	27.4	29.4	25.5
High school	65.9	64.8	66.9
Post-high school	6.8	5.9	7.7
Marital status			
Ever married	1.1	1.0	1.1
Never married	98.9	99.0	98.9
Toilet at home			
Yes	41.4	41.7	41.2
No	58.6	58.4	58.8
A private place to bathe and change at home			
Yes	38.1	45.0	31.4
No	61.9	55.0	68.6
Interpersonal practical guidance (IPG)			
Yes-Had a discussion on procurement	42.7	41.7	43.5
Yes-Had a discussion on use	44.3	42.8	45.8
Yes-Had a discussion on disposal	46.2	39.7	52.5
Knowledge			
Use-Correct changing	66.4	49.0	83.0
Disposal-Correct ultimate disposal	70.3	57.7	82.2
Attitudes			
Procure-Agree on embarrassment	53.5	54.5	52.5
Procure-Agree on inconvenience	44.4	47.0	42.0
Use-Agree on correct use for drying	78.3	78.4	78.3
Disposal-Agree on correct ultimate disposal	83.0	79.4	86.5
Practices			
Use-Correct storage	33.9	28.2	39.3
Use-Correct washing	56.6	48.3	64.6
Use-Correct drying	49.1	39.4	58.3
Disposal-Correct ultimate disposal	60.5	51.9	68.7

^a^ Adolescent girls in primary school and those who did not have any formal education are combined because less than 1.5% of adolescent girls did not have any formal education.

Chi-square analyses were conducted to explore the relationship between interpersonal and mediated practical guidance, sociodemographic and socioeconomic characteristics, and each KAP variable (Table 3). Additionally, unadjusted odds ratios were reported to explore the bivariate association between IPG and MPG status (no communication, IPG only, MPG only, and both), and their corresponding menstrual health related KAP measures. The comparison group with no IPG or MPG (None) was the reference group (Table 4).

**Table 3:** Distribution of respondents by type of interpersonal and mediated practical guidance (IPG and MPG) received and background characteristics^a^.

	Practical Guidance on Procurement				Practical Guidance on Use				Practical Guidance on Disposal			
	Comparison (%)		Intervention (%)		Comparison (%)		Intervention (%)		Comparison (%)		Intervention (%)	
	None (*n* = 677)	IPG Only (*n* = 485)	MPG Only (*n* = 690)	IPG = MPG (*n* = 532)	None (*n* = 665)	IPG Only (*n* = 497)	MPG Only (*n* = 662)	IPG = MPG (*n* = 560)	None (*n* = 701)	IPG Only (*n* = 461)	MPG Only (*n* = 581)	IPG = MPG (*n* = 641)
Age (years)												
12–14	27.0	20.2	28.0	19.7	27.4	19.9	28.0	20.2	26.8	20.2	29.4	19.8
15–17	43.3	51.8	47.5	47.0	45.0	49.3	47.7	46.8	45.4	49.0	45.8	48.7
18–19	29.7	28.0	24.5	33.3	27.7	30.8	24.3	33.0	27.8	30.8	24.8	31.5
District												
Jaunpur	34.7	36.5	37.5	37.2	34.1	37.2	37.5	37.3	35.0	36.2	39.6	35.4
Mirzapur	31.9	33.8	30.4	38.4	31.4	34.4	31.1	37.1	30.8	35.6	29.8	37.6
Sonbhadr^a^	33.4	29.7	32.0	24.4	34.4	28.4	31.4	25.5	34.2	28.2	30.6	27.0
Type of house												
Kucha	46.8	37.7	52.6	50.0	46.0	39.0	53.5	49.1	45.1	39.9	52.3	50.7
Semi pucca	23.3	27.8	21.7	25.0	21.7	30.0	20.4	26.4	24.0	27.1	22.7	23.6
Pucca	29.8	34.4	25.7	25.0	32.3	31.0	26.1	24.5	31.0	33.0	25.0	25.7
Education												
Primary school/ No formal education^b^	31.6	23.9	29.7	17.7	31.1	24.7	29.6	18.4	31.4	23.9	30.0	19.5
High school	63.0	69.4	63.4	73.3	63.3	68.8	64.6	71.5	63.9	68.5	63.9	71.2
Post-high school	5.4	6.7	6.9	9.0	5.5	6.5	5.8	10.1	4.8	7.7	6.1	9.3
Toilet at home												
Yes	38.0	46.8	39.3	43.8	37.9	46.7	40.0	42.7	38.8	46.0	37.5	44.6
Private place to bathe and change at home												
Yes	48.7	63.7	63.5	75.2	49.5	62.4	63.6	74.5	51.2	60.7	64.4	72.4

^a^ All results were statistically significant at *p* ≤ 0.05 level.

^b^ Adolescent girls in primary school and those who did not have any formal education are combined because less than 1.5% of adolescent girls did not have any formal education.

**Table 4:** Bivariate association between interpersonal and mediated practical guidance (IPG and MPG) received and their corresponding menstrual health related KAP measures.

	Knowledge OR (95% CI)		Attitudes OR (95% CI)				Practices OR (95% CI)			
	Correct use for changing	Correct ultimate disposal	Agree on embarrassment to procure	Agree on inconvenience to procure	Agree on correct use for drying	Agree on correct ultimate disposal	Correct storage use	Correct washing use	Correct drying use	Correct ultimate disposal
Practical Guidance on Procurement										
Comparison										
None^a^(*n* = 677)	—	—	Reference	Reference	—	—	—	—	—	—
IPG (*n* = 485)	—	—	1.2 (0.9–1.5)	0.9 (0.7–1.2)	—	—	—	—	—	—
Intervention										
MPG (*n* = 690)	—	—	1.1 (0.9–1.4)	0.8 (0.6–1.0)*^b^	—	—	—	—	—	—
IPG + MPG (*n* = 532)	—	—	1.2 (1.0–1.5)	0.8 (0.6–1.0)*^b^	—	—	—	—	—	—
Practical Guidance on Use										
Comparison										
None^a^ (*n* = 665)	Reference	—	—	—	Reference	—	Reference	Reference	Reference	—
IPG (*n* = 497)	1.1 (0.8–1.3)	—	—	—	0.8 (0.6–1.0)	—	1.2 (0.9–1.5)	2.0 (1.6–2.5)***	2.0 (1.6–2.5)***	—
Intervention								1.9		
MPG (*n* = 662)	5.0 (3.9–6.5)***	—	—	—	0.9 (0.7–1.2)	—	1.4 (1.1–1.8)**	(1.5–2.4)***	2.1 (1.7–2.7)***	—
IPG + MPG (*n* = 560)	5.5 (4.2–7.1)***	—	—	—	0.8 (0.6–1.1)	—	2.2 (1.7–2.8)***	4.0 (3.1–5.1)***	4.2 (3.3–5.4)***	—
Practical Guidance on Disposal										
Comparison										
None^a^ (*n* = 701)	—	Reference	—	—	—	Reference	—	—	—	Reference
IPG (*n* = 461)	—	1.8 (1.4–2.3)***	—	—	—	1.8 (1.3–2.4)***	—	—	—	2.0 (1.6–2.6)***
Intervention										
MPG (*n* = 581)	—	2.6 (2.0–3.2)***	—	—	—	1.4 (1.0–1.8)***^b^	—	—	—	1.5 (1.2–1.9)***
IPG + MPG (*n* = 641)	—	8.3 (6.1–11.1)***	—	—	—	3.4 (2.5–4.8)***	—	—	—	5.3 (4.2–6.9)***

^a^ The comparison group that did not receive IPG or MPG (“none”) is the reference group for all analyses.

^b^ Values were rounded to the nearest tenth. The original 95% CI upper bounds were MPG = 0.9925344 and IPG + MPG = 0.9615441 for the attitude variable related to practical guidance on procurement. The original 95% CI lower bound was IPG = 1.02051 for the attitude variable related to practical guidance on disposal.

* Statistical significance at *p* ≤ 0.05 level.

** Statistical significance at *p* ≤ 0.01 level.

*** Statistical significance at *p* ≤ 0.001 level.

Several multivariate logistic regressions were conducted controlling for sociodemographic and socioeconomic variables to determine the true association between interpersonal and mediated practical guidance and their KAP-related variables. The models examined the association between IPG and MPG status and the corresponding menstrual health related KAP variables. Results are reported using adjusted odds ratios (AOR) and are shown for each IPG-MPG status, with None (No IPG or MPG) as the reference group (Table 5).

**Table 5:** Multivariate analyses assessing the relationship between interpersonal and mediated practical guidance (IPG and MPG) and the corresponding KAP procurement, use, and disposal variables^a^.

	Knowledge AOR (95% CI)		Attitudes AOR (95% CI)				Practices AOR (95% CI)			
	Correct use for changing	Correct ultimate disposal	Agree on embarrassment to procure	Agree on inconvenience to procure	Agree on correct use for drying	Agree on correct ultimate disposal	Correct storage use	Correct washing use	Correct drying use	Correct ultimate disposal
Practical Guidance on Procurement										
Comparison										
None^b^ (*n* = 677)	—	—	Reference	Reference	—	—	—	—	—	—
IPG (*n* = 485)	—	—	1.2 (0.9–1.5)	1.0 (0.7–1.2)	—	—	—	—	—	—
Intervention										
MPG (*n* = 690)	—	—	1.1 (0.9–1.4)	0.8 (0.7–1.0)	—	—	—	—	—	—
IPG + MPG (*n* = 532)	—	—	1.2 (1.0–1.6)	0.8 (0.7–1.1)	—	—	—	—	—	—
Practical Guidance on Use										
Comparison										
None^b^ (*n* = 665)	Reference	—	—	—	Reference	—	Reference	Reference	Reference	—
IPG (*n* = 497)	1.1 (0.8–1.4)	—	—	—	0.7 (0.5–1.0)*^c^	—	1.0 (0.8–1.4)	2.2 (1.7–2.8)***	2.0 (1.6–2.6)***	—
Intervention										
MPG (*n* = 662)	5.4 (4.1–7.0)***	—	—	—	1.0 (0.7–1.2)	—	1.5 (1.1–1.9)**	2.0 (1.6–2.5)***	2.2 (1.7–2.7)***	—
IPG + MPG (*n* = 560)	5.5 (4.1–7.3)***	—	—	—	0.8 (0.6–1.1)	—	2.0 (1.6–2.6)***	4.3 (3.3–5.5)***	4.4 (3.4–5.7)***	—
Practical Guidance on Disposal										
Comparison										
None^b^ (*n* = 701)	—	Reference	—	—	—	Reference	—	—	—	Reference
IPG (*n* = 461)	—	1.8 (1.4–2.3)***	—	—	—	1.8 (1.3–2.5)***	—	—	—	2.1 (1.6–2.6)***
Intervention										
MPG (*n* = 581)	—	2.8 (2.2–3.6)***	—	—	—	1.4 (1.0–1.8)*^c^	—	—	—	1.6 (1.3–2.0)***
IPG + MPG (*n* = 641)	—	8.6 (6.3–11.8)***	—	—	—	3.3 (2.4–4.6)***	—	—	—	5.4 (4.2–7.1)***

^a^ Controlling for sociodemographic and socioeconomic characteristics.

^b^ The comparison group that did not receive IPG or MPG (“none”) is the reference group for all analyses.

^c^ Values were rounded to the nearest tenth. The original 95% CI upper bound was IPG = 0.995241 for the attitude variable related to practical guidance on use. The original 95% CI lower bound was IPG = 1.02051 for the attitude variable related to practical guidance on disposal.

* Statistical significance at *p* ≤ 0.05 level.

** Statistical significance at *p* ≤ 0.01 level.

*** Statistical significance at *p* ≤ 0.001 level.

Relationships were statistically significant at a *p*-value less than or equal to 0.05. For regression analysis, if the 95% confidence interval included 1, the predictor was not considered significant. Though religion and marital status were included in the questionnaire, they were excluded from the multivariate analyses because of the homogeneity of responses for those characteristics.

## 4. Results

Descriptive statistics in Table 2 show adolescent girls' sociodemographic and socioeconomic characteristics for the intervention and comparison groups. Overall, the respondents were equally distributed across all three districts in Uttar Pradesh, India (36.5% in Jaunpaur, 33.3% in Mirzapur, 30.2% in Sonbhadra), and 97.2% practiced Hinduism, and 98.9% were never married. Nearly half of all adolescent girls (47.1%) were between 15 and 17 years of age, and almost two-thirds of all respondents were currently in high school (65.9%). Just under half of all respondents lived in raw or temporary housing (kucha) (47.4%), although 61.9% did have a separate place to bathe and change at home. However, 58.6% did not have a working toilet at home. Intervention and comparison groups were matched on sociodemographic and socioeconomic characteristics, which explains their similarity. This allowed for appropriate comparison in the study.

IPG on the procurement and use of menstrual products were similar among the intervention and comparison groups. However, a higher proportion of adolescent girls in the intervention group received IPG on the disposal of menstrual products (52.5% vs. 39.7%). Additionally, attitudes towards the procurement and use of menstrual products were similar across all groups. However, a higher proportion of adolescent girls in the intervention group reported agreeable attitudes towards the correct ultimate disposal of products (86.5% vs. 79.4%). Lastly, a higher proportion of intervention group girls reported correct knowledge and practices on the use and disposal of menstrual products compared to the comparison group.

Table 3 examines adolescent girls' sociodemographic and socioeconomic characteristics and their IPG and MPG status. There are no substantial differences by age, district, and type of house. Girls in the intervention arm who received both IPG and MPG on the procurement, use, and disposal of products, however, appear to be more educated, and more likely to have a private bathe and place to change at home, especially compared with girls who had neither (None group). For instance, over 70% of girls who received IPG and MPG on each KAP area had a high school education, whereas approximately 63% of girls who received neither had the same level of education. Also, among girls with IPG and MPG on the procurement, use, and disposal of products, about three-quarters had access to a private bathing and changing place compared to only half of the girls who received no IPG or MPG.

The unadjusted bivariate results in Table 4 show significant associations between IPG and MPG status and the corresponding KAP variables on the procurement, use, and disposal of menstrual products. Adolescent girls who received no IPG or MPG (“none”) were used as the reference group.

Practical guidance on the use of menstrual products is not associated with KAP attitudes on correct drying of washed menstrual cloth. However, while IPG on use is not significantly associated with KAP knowledge related to how often to change menstrual pads/cloth in a day, MPG on use is significantly associated with this KAP knowledge. Indeed, the similar odds ratio for MPG only [OR: 5.0 (95% CI, 3.9–6.5)] and both IPG and MPG [OR: 5.5 (95% CI, 4.2–7.1)] is consistent with the no effect observed for IPG among the control group. This suggests that the positive effect observed among the intervention group comes from MPG only.

The receipt of practical guidance on using menstrual products was also significantly associated with correct KAP practices for adolescent girls. Among those who received only MPG, they had 1.4 (95% CI, 1.1–1.8) times the odds of reporting correct storage practices compared to girls who received no form of practical guidance, and those that received IPG and MPG had 2.2 (95% CI, 1.7–2.8) times the odds. Moreover, girls who received only IPG or MPG had similar odds of reporting correct washing and drying practices compared to girls who did not receive anything. Those with IPG and MPG had 4.0 (95% CI, 3.1–5.1) times greater odds of reporting correct washing and 4.2 (95% CI, 3.3.1–5.4) times greater odds of correct drying practices.

Finally, among girls who received IPG, MPG, or both forms of practical guidance on disposal of menstrual products, there were significant associations with the corresponding KAP variables. Adolescent girls who received only IPG had a 1.8 (95% CI, 1.4–2.3) times greater odds of reporting correct KAP knowledge on the ultimate disposal of menstrual products compared to girls that received no practical guidance. Girls who received only MPG had 2.6 (95% CI, 2.0–3.2) times the odds. However, those with both IPG and MPG had 8.3 (95% CI, 6.1–11.1) times the odds of having the correct knowledge on the ultimate disposal of products. Regarding adolescent girls' KAP attitudes related to ultimate disposal of menstrual products, those with IPG alone or MPG alone had 1.8 (95% CI, 1.3–2.5) and 1.4 (95% CI, 1.0–1.8) times the odds of agreeing with the correct ultimate disposal of sanitary products, respectively, compared to girls who received no practical guidance. However, girls with both IPG and MPG had 3.4 (95% CI, 2.5–4.8) times the odds compared to girls who received no practical guidance. For correct KAP practices related to ultimate disposal, the respective odds ratios for those with only IPG, only MPG, or IPG and MPG are 2 (95% CI, 1.6–2.6), 1.5 (95% CI, 1.2–1.9), and 5.3 (95% CI, 4.2–6.9) compared to girls who received no practical guidance.

These relationships are further investigated in Table 5 controlling for a range of sociodemographic and socioeconomic variables. The results are unchanged from the bivariate associations, suggesting that the effect of IPG and MPG on KAP related to procurement, use, and disposal of menstrual products are not affected by the various sociodemographic and socioeconomic factors. The only difference is that the significant effect of MPG on KAP attitudes related to inconvenience of procuring sanitary products became insignificant.

## 5. Discussion

This study aimed to answer two research questions: how IPG and SBCC-driven MPG independently influence adolescent girls' KAP on the procurement, use, and disposal of menstrual products and whether those who receive both IPG and MPG have better KAP related to the procurement, use, and disposal of menstrual cloth or sanitary pads than those who receive one form of practical guidance or none at all.

To begin, the study's findings show that girls who receive both IPG and MPG tend to be more educated, especially when compared with girls who did not receive any form of practical guidance. Current literature cites the importance of school-based MHHM education for girls (2, 36) including the potential to address negative attitudes towards menstruation (36). This education is essential because girls have reported being unaware and with feelings of shock, fear, anxiety, guilt, and frustration at menarche (9). Additionally, girls who received both IPG and MPG also were more likely to have a private place to bathe and change at home when compared with those who received neither. These disparities highlight the physical barriers that adolescents may face while managing their menstruation. Otherwise, there were no substantial differences regarding each KAP area and sociodemographic and socioeconomic characteristics such as age, district, and type of house.

The results also show that practical guidance on procurement is ineffective in affecting KAP attitudes related to the procurement of sanitary products. More than three-quarters of all adolescent girls in the study agree it is inconvenient to procure sanitary products, and more than half agree it is embarrassing. The original SBCC evaluation study published in 2018 also reported that the intervention did not adequately address all their attitude variables (8). Attitudes may be more difficult to influence because they are the affective evaluation of behavior (19) and relate to personal emotion and normative practices. Attitudes could also be shared by all adolescent girls and their support systems, regardless of group, and subtly influence the sender and receiver's delivery and receipt of practical guidance. Unlike practical guidance on correct menstrual health knowledge and practices, attitudes are less clear subject matter. Consequently, attitudes might require more investigation into how IPG is being delivered in addition to if it is occurring. This understanding is essential because how information is communicated influences attitudes (20). Moreover, attitudes can help predict whether that behavior takes place (19).

There is an opportunity for IPG and MPG on procurement to tackle the issue of adolescent girls feeling embarrassed to procure sanitary products. Communication strategies have been identified as promising ways to change attitudes on menstrual health alongside increasing correct knowledge and practices (14–16). One UNICEF-led SBCC intervention that utilized interpersonal and mediated communication strategies in Maharashtra state saw changes in attitudes related to embarrassment, fear, and humiliation (17). Specifically, they found that mothers who became key sources of correct information helped shift attitudes, stimulate household conversation, and provide daughters with menstrual products. Breaking the culture of silence around menstruation can help make adolescents less embarrassed to procure sanitary products. Additionally, practical guidance must include correct information sharing that empowers women and girls, increases positive attitudes, and translates into action.

Regarding adolescent girls feeling that it is inconvenient to procure sanitary products, it is unclear what this means. To the extent that this inconvenience refers to financial or physical barriers, such as the inaccessibility of products, it is understandable that practical guidance, which focuses more on providing information, would be an ineffective strategy to address this gap. In the UNICEF-led intervention in Maharashtra state, they also highlighted the importance of understanding these barriers and discussed the importance of addressing these alongside improving KAP (17).

For practical guidance on use, the results show that IPG is ineffective with respect to KAP knowledge on how many times to change a menstrual product in a day. However, those with MPG have significantly greater odds of reporting correct knowledge [AOR: 5.4 (95% CI, 4.1–7.0)]. While IPG refers to face-to-face discussions which could come from their support network, parents and others in this network may not necessarily have the correct knowledge themselves. In contrast, MPG comes from experts who provide evidence-based information. Thus, adolescent girls receiving practical guidance from mediated communication sources may have much better knowledge about how often to change sanitary products.

For KAP practices, the effect of practical guidance on use appears to be additive. For example, in relation to KAP practices on the correct ultimate disposal of products, receiving one form of practical guidance- only IPG or only MPG- is significantly associated with an adjusted odds ratio of 2.0 (95% CI, 1.6–2.6)–2.2 (95% CI, 1.7–2.7), respectively, compared to receiving neither. However, receiving both doubles the AOR to 4.3 (95% CI, 3.3–5.5)–4.4 (95% CI, 3.4–5.7) compared to receiving none. This may suggest that the pathways of influence of IPG and MPG on KAP practices are different and complement each other.

Concerning practical guidance on the disposal of sanitary products, receiving both IPG and MPG results in much superior KAP on the disposal of menstrual products. Those who received IPG and MPG on the disposal of products had far greater odds of reporting better KAP when compared to girls who only received MPG, IPG, or nothing at all. One of the reasons IPG on the disposal of products was most notable may be that it was a newer topic, which stimulated more discussion on this topic. It is also possible that since the majority of adolescent girls in this study were 15–17 years old, they may have already reached menarche, and as a result, had previously discussed procurement, making them less inclined to continue to seek guidance on menstrual product procurement.

Other studies have championed IPC or IPC alongside mediated communication as mechanisms to empower women and girls, include men and boys, and improve menstrual health and management in India (15, 27). A study on menstrual health IPC among adolescents in rural, tribal, and urban communities in Western India found that “ever asking about menstrual health” was associated with positive beliefs and knowledge (27). Another community-based intervention in the Wardha district of Maharashtra state that involved adolescent girls' forums in raising awareness of MHHM demonstrated the utility of print materials during monthly meetings to communicate key messages and created sustained behavior changes in menstrual management (37). Lastly, a school-based menstrual health peer educator program among female adolescents in Keru, Jodhpur, Rajasthan successfully used peer-to-peer IPC and mediated communication to increase KAP (36). The program evaluation further emphasized the importance of repetition of messages through intervention components (i.e., peer-led group sessions) to create and sustain engagement.

India's government has also supported and prioritized interpersonal and mediated communication for MHHM programs (14). For instance, “Swachh Bharat Abhiyan” (Clean India Mission) is a country-wide government program to achieve universal sanitation (38), and since 2015, this program has incorporated MHHM (15). Moreover, the Ministry of Drinking Water and Sanitation in India developed an Advocacy and Communication Strategy Framework in collaboration with UNICEF and other partners that included interpersonal and mediated communication as critical components to addressing hygiene and sanitation issues at the national, state, and district levels (14). This governmental support and prioritization of multi-pronged communication strategies and MHHM offer opportunities to advance interpersonal and mediated communication as mechanisms to deliver practical guidance on menstrual management and allow for programs to further highlight the additive effect of IPG combined with MPG.

These innovative ways to deliver practical guidance are a foundational step to addressing factors that may impede the achievement of effective menstrual management. Moreover, while this study found IPG or MPG delivered independently do predict better KAP that can help achieve MHHM, the delivery of both forms of practical guidance as part of a strategic SBCC intervention is particularly beneficial for adolescent girls' MHHM. With these favorable steps, we are closer to attaining adolescent girls' human right to adequate MHHM.

## 6. Limitations

There are several limitations to this study. Practical guidance on menstrual management often includes communication on pain management and when to seek care (1, 6). However, the original questionnaire was not structured to answer this study's specific research questions and therefore did not contain questions on menstrual pain or care-seeking. For this reason, it was not possible to examine those topics in relation to interpersonal and mediated practical guidance. Similarly, the procurement, use, and disposal of products were not addressed under each variable related to knowledge, attitudes, and practices. Consequently, procurement is only examined by two attitude questions because there were no relevant knowledge or practice questions. Lastly, MPG within the intervention could not be individually assessed because of the inability to identify the specific activities that provided mediated communication on the procurement, use, and disposal of menstrual products.

## 7. Conclusion

MHHM is a complex human rights and gender equity issue that has seen increased recognition by global and local communities. However, the culture of silence surrounding menstruation in India exacerbates systematic factors and impedes progress. Addressing this silence requires innovation, which is crucial to improving MHHM in India as there is a lack of effective SBCC strategies and materials to address this issue (15).

GARIMA demonstrated success in breaking this silence with measured increase in interpersonal communication (8), however, it did not assess the role of interpersonal communication as a mechanism for practical guidance. Moving forward, future studies must seek to understand this role for interpersonal and mediated communication as mechanisms for practical guidance. Accordingly, the next steps are to design and validate an instrument to effectively capture key practical guidance components related to menstrual management KAP and implement this tool during an SBCC intervention where IPG and MPG are delivered together. The instrument should also seek to capture how practical guidance is being provided rather than solely focusing on whether it is occurring. It is possible that interventions focus on providing the appropriate information and increasing communication but are not paying sufficient attention to how family members, teachers, or peer educators communicate this information to others. This absence may decrease the intervention's effectiveness. Alternatively, giving attention to this may help to better understand attitudes towards menstrual health management. Moreover, future interventions must also consider who delivers the practical guidance, how they can provide the necessary skills to provide practical guidance in a timely, accurate, and comfortable manner, and what barriers and facilitators are being faced within the specific context related to the socioecological levels.

Now is the time to address MHHM in India and around the globe through innovative strategies, such as interpersonal and mediated practical guidance. Every individual deserves the right to safety, dignity, and respect. With the increased recognition, there are increasing opportunities to employ these strategies to promote adequate MHHM. Thus, despite the challenges and cultures of silence, the current climate provides an opportunity to create positive, sustainable changes, and it begins with ensuring all women and girls are prepared to confidently manage their menstrual health.

## Data Availability

The datasets analyzed for this study will be made available by the corresponding author upon request.
